# A Clinicopathological Report of Four Cases of Squamous Odontogenic Tumor-Like Proliferations in Odontogenic Cysts: Suggested Opinions regarding This Unusual Nature

**DOI:** 10.1155/2020/6978587

**Published:** 2020-02-14

**Authors:** Massoumeh Zargaran, Setareh Shojaei

**Affiliations:** ^1^Department of Oral and Maxillofacial Pathology, Faculty of Dentistry, Kurdistan University of Medical Sciences, Sanandaj, Iran; ^2^Department of Oral& Maxillofacial Pathology, School of Dentistry, Hamadan University of Medical Sciences, Hamadan, Iran

## Abstract

Distinguishing squamous odontogenic tumor-like proliferations (SOTLPs) is important in odontogenic cysts to avoid misinterpretation such as a squamous odontogenic tumor, well-differentiated squamous cell carcinoma, and acanthomatous type of ameloblastoma. This study is aimed at reporting 4 cases of these clinicopathological proliferations in order to shed more light on the importance of distinguishing them from other similar types. 150 odontogenic cysts were studied in which four cases (2.66%) with SOTLPs were identified including 2 radicular cysts, 1 dentigerous cyst, and 1 odontogenic keratocyst. These proliferations were observed in the cysts' wall particularly adjacent to the epithelial lining. All cysts had inflammation while 3 cases showed budding from the epithelial cyst lining. The findings suggested that lining of odontogenic cysts could be a source of SOTLPs, and inflammation probably played an effective role in their development. Its incidence was 2.66% in the present study. Although SOTLPs are not frequent in odontogenic cysts, their identification is important to prevent wrong histopathologic interpretation and treatment.

## 1. Introduction

Squamous odontogenic tumor (SOT) is a rare benign neoplasm with odontogenic epithelium origin. SOT growth is slow/gradual, but it might invade the trabecular bone and infiltrate into its adjacent tissues. This tumor histopathologically is described as the proliferation of well-differentiated squamous epithelial cells that create islands and nests located in a fibrous stroma [1]. SOT should be differentiated histopathologically from a similar feature called a squamous odontogenic tumor-like proliferation (SOTLP) [1–3].

SOTLPs are a rare phenomenon [2, 4]. These proliferations occur in the walls of odontogenic cysts [1, 3, 5, 6] while they are morphologically similar to epithelial islands that form SOT [1–3]. In spite of this similarity, there is no evidence of neoplastic transformation [1–3]; in fact, its biological behavior and prognosis is different from SOT [2, 3, 7]. Histopathological appearance of SOTLPs overlaps with that of some other neoplasms like the acanthomatous type of ameloblastoma and the well-differentiated squamous cell carcinoma (SCC) [1–3]. Due to this similarity in microscopic appearance, SOTLPs can be misinterpreted by a pathologist who is not aware of the subtle differences between this phenomenon and other stated lesions [2–4]. The present study is aimed at reporting 4 cases of odontogenic cysts with SOTLPs and comparing them with other reports in this regard.

## 2. Case Report

150 odontogenic cysts (inflammatory or developmental) belonging to the archive of the Department of Oral Pathology, Faculty of Dentistry, Hamadan University of Medical Sciences, were studied using an optical microscope. All hematoxylin and eosin (H&E) slides were carefully inspected for presence of SOTLPs. Presence of several squamous epithelial islands that were morphologically similar to SOT was considered as a histopathological criterion for diagnosing SOTLPs [3].

**Figure 1:**
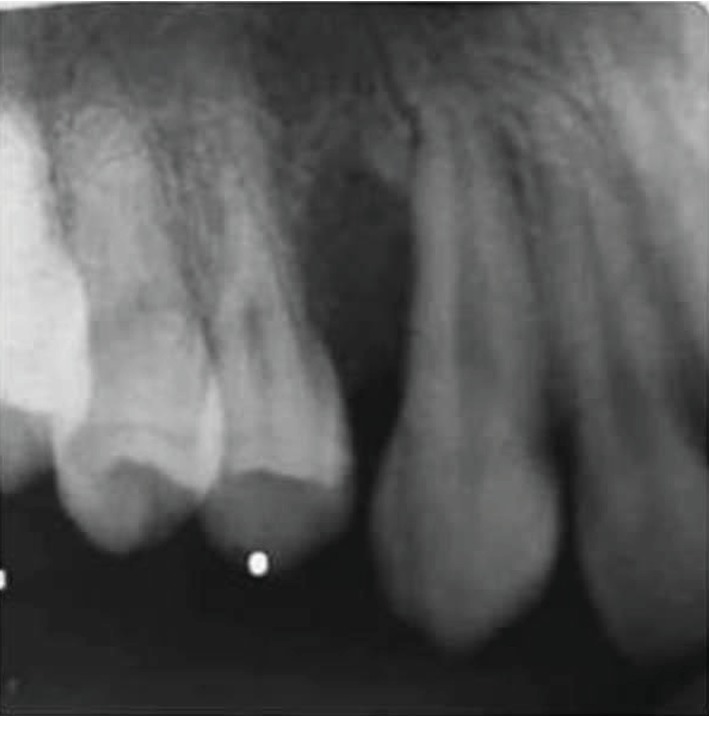
Radiography image of a unilocular, cystic radiolucent lesion between the maxillary first premolar and canine.

Clinical information was obtained from the patients' records. Four cases including 2 radicular cysts, 1 dentigerous cyst, and 1 odontogenic keratocyst (OKC) were detected with SOTLPs; three cases were in men (75%) and one in a woman (25%). All patients were white, between 18 and 28 years old, and 2 cysts were in the maxilla and 2 in the mandible. Radiographical appearances of the lesions in the records of the patients were described as unilocular, cystic, or well-circumscribed radiolucency (Figure 1). These radiographical appearances and the records of intraoperative findings for all samples were interpreted as cystic lesions, and gross descriptions of them were matched to a cystic nature.

**Figure 2:**
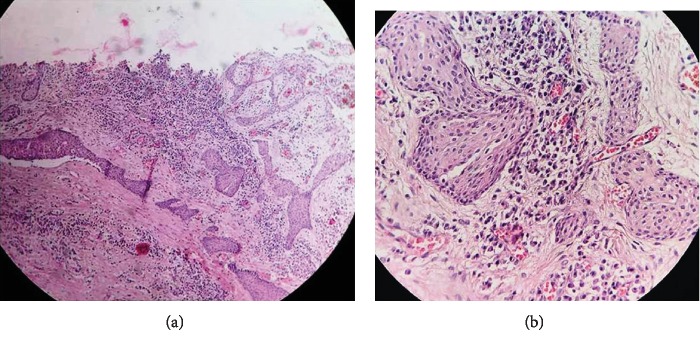
(a) Islands of various sizes consisting of well-differentiated squamous cells with bland appearance within the connective tissue wall of the cyst; H&E, ×10. (b) Islands of various sizes consisting of well-differentiated squamous cells with bland appearance within the connective tissue wall of the cyst; H&E, ×40.

**Figure 3:**
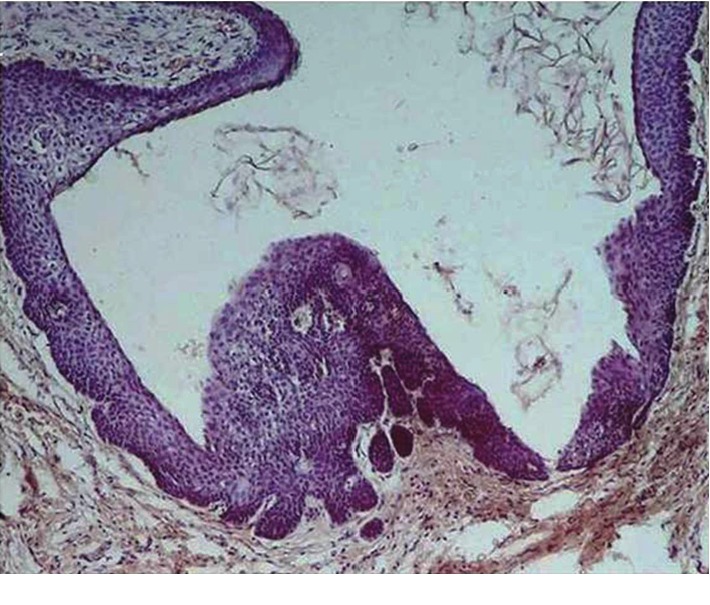
Budding from the epithelial lining of OKC; H&E, ×10.

**Figure 4:**
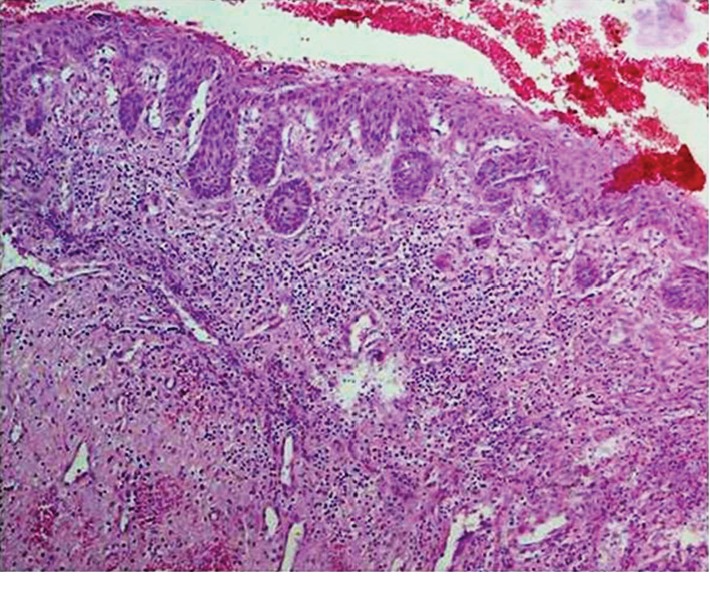
Budding from the epithelial lining of the radicular cyst; H&E, ×10.

Histopathologically, SOTLPs were observed as islands in various sizes consisting of well-differentiated squamous cells with bland appearance within the connective tissue wall of the cyst especially adjacent to the cyst lining (Figures 2(a) and 2(b)). The peripheral cells of these islands were flat, and some were cubic basaloid cells. Dyskeratotic cells, keratin pearl formation, microcyst formation, and mitosis were not observed in any of the islands. All detected cysts had inflammatory components as primary or secondary in nature. Changes such as hyperplasia, desquamation, and/or lining replacement with granulation tissue were observed in the epithelial lining of lesions. The inflammation type of the cysts was chronic, and it mainly consisted of lymphocytes and/or plasma cells. Degree and intensity of the inflammations were mild to moderate and were dispersed. Budding from the epithelial lining was observed in 3 cases (Figures 3 and 4), and 1 case represented nests and epithelial cords consistent with rests of Malassez.

## 3. Discussion

Almost 60 odontogenic cysts with STOLPs were reported in English language articles searched in PubMed and MEDLINE particularly by Sala-Pérez et al. [1–3, 5–9]. These four cases found in the present study should be added to previously reported cases. In fact, there are only a few case series reporting STOLPs, most of which reported only one case, and their clinicopathological information did not seem to be adequate when compared with the present study [1, 7–9] except for Parmar et al. [2] and Wright [3].

Out of 150 odontogenic cysts investigated in the present study, 4 cases were found with SOTLPs (2.66%). The number of cases reported by Parmar et al. was more than that in this study; their percentage of SOTLPs cases was 3.4%. The range of patients' age in the Parmar et al. and Wright studies [2, 3] was 28-80 and 31-65 years old, respectively, whereas it was 18-28 for the current study. Histopathologically, no pearl keratin, dyskeratotic cells, and microcyst formation were observed in epithelial islands in this study unlike Parmar et al. [2]. However, absence of mitosis and presence of chronic inflammation mostly formed of lymphocytes and/or plasma cells were similar to Parmar et al. [2]. Degree of inflammation was mostly moderate to severe for Parmar et al. [2], but it was mild to moderate for this study although inflammation distribution was diffused for both. Budlike extensions and presence of rest of Malassez were observed in some samples of both studies too.

SOTLPs are rare histopathological findings [2, 4] with unknown etiology [1, 4]. However, there are different opinions on epithelial origin of SOTLPs including the following:

- The odontogenic epithelial remnants particularly the rest of Malassez could be proposed as an important source of such epithelial proliferations [3, 4] because of (a) the proliferative ability of these remnants which apparently had an important role in the pathogenesis of odontogenic cysts, (b) their squamous differentiation in some of the cases [4], and (c) the closeness of the cysts with SOTLPs to the root of the teeth [3]. Islands and epithelial cords with inactive appearance consistent with the rest of Malassez were observed in 1 case of this study and 4 cases reported by Parmar et al. [2] with no evidence of hyperplasia or squamous differentiation
- Inflammatory reactive hyperplasia of cyst lining was also mentioned as STOLPs' origin [10]. In this study, STOLPs were adjacent to the epithelial lining of cysts which became hyperplastic or desquamated and was replaced with granulation tissue. They were observed in the cases of the dentigerous cyst and OKC in the regions whose epithelial lining had neither typical morphology nor diagnostic criteria. In Wright's study, 3 of 4 dentigerous cysts had inflammation and the histopathological appearance of the epithelial lining was not typical [3]
- Another suggestion was hyperplasia with the form of budding in the epithelial lining of radicular cysts in response to a subsided inflammation, because these budlike extensions usually occurred in areas without inflammation and they were histopathologically similar to the squamous epithelial islands in the cyst wall [2]. However, the buddings were found in radicular cysts and OKC with/without subsided inflammation in the present study. Hence, it seemed that the buddings in the epithelial lining necessarily were not a consequence of subsided inflammation

Moreover, this study found that hyperplasia and budding in the epithelial lining of the cyst might be the direct origin of SOTLPs. Even in cases where the epithelium lining was desquamated or replaced with granulation tissue, these changes seemed to have occurred in the lining after budding and separation of squamous odontogenic tumor like islands. Consequently, the source of epithelial lining of odontogenic cysts could be considered as the indirect origin of SOTLPs.

In addition to that, the role of inflammation in SOTLPs seemed to be unknown [2]. Parmar et al. [2] suggested that, due to finding SOT-like islands in inflammation-free areas, inflammation might not be the initial stimulus for SOTLPs in radicular cysts. The results also revealed the role of inflammation as the stimuli of SOTLPs although it was not considered as the only definitive cause of these proliferations since few samples were found with SOTLPs. Considering that SOTLPs were present in only four cases of primary/secondary inflammatory odontogenic cysts in this study, there might be other unknown factors influencing this phenomenon.

In fact, SOTLPs were morphologically similar to SOT although they did not seem to have any tendency to develop a solid tumor [1–3]. Like Wright's study [3], all samples of the current study had radiographically a provisional diagnosis of a cyst, and they were all cystic in terms of gross description.

Care must be taken that the overlapping histopathological features of SOTLPs, ameloblastoma (especially the acanthomatous type), and well-differentiated SCC do not lead to misdiagnosis. Ameloblastoma typically has islands with peripheral columnar ameloblast-like cells which have a clear cytoplasm and nucleus reverse polarity [2]. In this study, the peripheral cells of all islands were flat with eosinophilic cytoplasm to cuboidal basaloid with little cytoplasm. Budlike extensions off the epithelial lining of the cyst and the presence of islands of squamous cells adjacent to the lining made the lesions so similar to islands of SCC. Therefore, they could be mistaken for a primary intraosseous SCC arising in an odontogenic cyst. The SCC islands have cytologic atypia including altered nuclear cytoplasmic ratios, nuclear hyperchromatism, and mitotic activity [2]. In present study, the islands were free of any malignant features such as cellular atypia seen in SCC.

## 4. Conclusion

The findings of this study suggested that SOTLPs likely originated from the odontogenic cyst lining and inflammation might play a role for their development. STOLPs were considered as a rare histopathological finding, and their incidence was 2.66% in the present study. Although SOTLPs were not frequent in odontogenic cysts, their diagnosis was very important. The pathologists' lack of knowledge and their neglecting the specific features of SOTLP may lead to histopathological misinterpretation and consequently an improper treatment. As a result, the sophisticated and subtle differences of SOTLPs must be carefully understood to distinguish them from other lesions with similar appearance in order to avoid radical and unnecessary surgeries.
